# Supplementary material for: Germination and seedling establishment for hydroponics: The benefit of slant boards
Source: PLoS One. 2022 Oct 5;17(10):e0275710. doi: 10.1371/journal.pone.0275710 (PMC9534409; doi:10.1371/journal.pone.0275710)
Supplement: S1 File — (PDF) [file pone.0275710.s001.pdf]

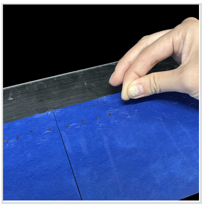

Germination and seedling establishment for deep-flow hydroponics: The benefit of slant boards V.2

Version 2

Aug 12, 2022

noah.langenfeld[1], Bruce Bugbee[1]

[1]Utah State University

1 Works for me Share

dx.doi.org/10.17504/protocols.io.5jyl89b86v2w/v2

USU Crop Physiology Laboratory

noah.langenfeld

ABSTRACT

Germination and seedling establishment for transplanting into hydroponics often uses porous substrates, but fine roots grow into these substrates, and they cannot be removed without damaging roots. Seedlings transplanted without removal of substrates can cause interactions with solution chemistry or addition of particulates to the nutrient solution. Germination of seeds on slant boards is clean, uniform, and reduces the time to transplanting. Slant boards facilitate long roots, which maximize exposure of the primary root to the nutrient solution after transplanting. The "boards" are made from thin acrylic or polycarbonate sheets with germination paper on top. Seeds are held in place by covering with thin paper before vertical placement of the boards in a container. Four to twelve days later, the seedlings with long roots can be removed from the paper without damage and transplanted into the hydroponic system. Here we describe slant board construction and procedures for rapid germination and transplanting.

DOI

dx.doi.org/10.17504/protocols.io.5jyl89b86v2w/v2

PROTOCOL CITATION

noah.langenfeld, Bruce Bugbee 2022. Germination and seedling establishment for deep-flow hydroponics: The benefit of slant boards. **protocols.io**
https://protocols.io/view/germination-and-seedling-establishment-for-deep-fl-cbpusmnw
Version created by noah.langenfeld

Citation: noah.langenfeld, Bruce Bugbee Germination and seedling establishment for deep-flow hydroponics: The benefit of slant boards https://dx.doi.org/10.17504/protocols.io.5jyl89b86v2w/v2

This is an open access protocol distributed under the terms of the **Creative Commons Attribution License** (https://creativecommons.org/licenses/by/4.0/), which permits unrestricted use, distribution, and reproduction in any medium,

FUNDERS ACKNOWLEDGEMENT

NASA
Grant ID: NNX17AJ31G

LICENSE

This is an open access protocol distributed under the terms of the Creative Commons Attribution License, which permits unrestricted use, distribution, and reproduction in any medium, provided the original author and source are credited

CREATED

Jun 20, 2022

LAST MODIFIED

Aug 12, 2022

PROTOCOL INTEGER ID

64980

MATERIALS TEXT

- Seeds
- 2 mm thick acrylic board
- Germination blotter paper
- Single-ply cellulose paper (Kimwipes)
- Acrylic holder
- Exterior container (plastic storage tote)
- Water

Seeding

1 Clean a rigid plastic slant board and lay flat on a clean surface.

2 Cut a piece of germination paper 10 cm in height and the same length as the slant board.

3 Place germination paper on top of slant board.

Citation: noah.langenfeld, Bruce Bugbee Germination and seedling establishment for deep-flow hydroponics: The benefit of slant boards https://dx.doi.org/10.17504/protocols.io.5jyl89b86v2w/v2

This is an open access protocol distributed under the terms of the **Creative Commons Attribution License** (https://creativecommons.org/licenses/by/4.0/), which permits unrestricted use, distribution, and reproduction in any medium,

4 Place seeds on top of germination paper (about 9 cm from the bottom of the board and 1 cm from the top of the germination paper) in a line parallel to the top and bottom of the slant board. Seeds should be about 5 mm apart.

5 Place a single layer of thin absorbent paper (Kimwipe) over the seeds.

6 Use a squirt bottle to gently wet the entire germination board and thin absorbent paper with water to lock seeds and paper in place on the board. Do not use nutrient solution.

7 Transfer the slant board into the exterior container. The angle of the board should be between 70° and 80°.

8 Fill the exterior container with purified water with calcium until the water level is about 2 cm above the bottom of the germination paper.

Monitoring and transplanting

9 After at least one cotyledon leaf has appeared, gently peel back the thin absorbent paper to expose the leaves. The thin absorbent paper should remain covering the roots.

10 After the roots have grown long enough to reach the solution, remove the seedlings from the paper. Fingers or a tweezers can be used to remove the seedlings. Avoid excessive pressure to prevent root damage.

11 Place the seedling in a neoprene collar. Ensure the roots are not pinched in the collar. The cotyledons can be used to support the seedling and stop it from falling into the solution.

12 Place the neoprene collar with the seedling into the main culture tank. Ensure to roots can reach the nutrient solution to avoid desiccation.

Citation: noah.langenfeld, Bruce Bugbee Germination and seedling establishment for deep-flow hydroponics: The benefit of slant boards https://dx.doi.org/10.17504/protocols.io.5jyl89b86v2w/v2

This is an open access protocol distributed under the terms of the **Creative Commons Attribution License** (https://creativecommons.org/licenses/by/4.0/), which permits unrestricted use, distribution, and reproduction in any medium,
