# Supplementary material for: Germination and seedling establishment for hydroponics: The benefit of slant boards
Source: PLoS One. 2022 Oct 5;17(10):e0275710. doi: 10.1371/journal.pone.0275710 (PMC9534409; doi:10.1371/journal.pone.0275710)
Supplement: S2 File — (DOCX) [file pone.0275710.s002.docx]

**S2 File. Root length measurements from Figure 11 in main text.**

Table 1: Length of lettuce cv. Grand Rapids roots over time.

| Lettuce seed number | ------------------------------------Root length (mm) ------------------------------------ | | | | | | | |
| --- | --- | --- | --- | --- | --- | --- | --- | --- |
|  | Day 0 | Day 1 | Day 2 | Day 3 | Day 4 | Day 5 | Day 6 | Day 7 |
| 1 | 0 | 0 | 0.5 | 1.4 | 1.5 | 2.1 | 2.4 | 2.7 |
| 2 | 0 | 0 | 0.3 | 1.5 | 2.2 | 2.9 | 3.3 | 3.9 |
| 3 | 0 | 0 | 0 | 0.4 | 0.9 | 1.6 | 2.1 | 2.5 |
| 4 | 0 | 0 | 0 | 0 | 0 | 0 | 0 | 0 |
| 5 | 0 | 0 | 0.5 | 1.6 | 2.2 | 3.2 | 3.9 | 4.4 |
| 6 | 0 | 0 | 0.5 | 1.3 | 1.6 | 2.2 | 3 | 3.8 |
| 7 | 0 | 0 | 0 | 0.5 | 1.3 | 2 | 3.5 | 3.9 |
| 8 | 0 | 0 | 0.4 | 1.6 | 2.2 | 2.1 | 3.4 | 4 |
| 9 | 0 | 0 | 0.7 | 0.9 | 1.2 | 0.6 | 0.6 | 1 |
| 10 | 0 | 0 | 0.7 | 1.9 | 3.2 | 4.3 | 6 | 6.2 |
| 11 | 0 | 0 | 0.4 | 1.3 | 1.6 | 2.3 | 3.3 | 3.4 |
| 12 | 0 | 0 | 0.4 | 1.7 | 3.2 | 3 | 3.2 | 3.2 |
| 13 | 0 | 0 | 0 | 0.3 | 1 | 2 | 3.5 | 4.4 |
| Average | 0 | 0 | 0.3 | 1.1 | 1.7 | 2.2 | 2.9 | 3.3 |
| Standard deviation | 0 | 0 | 0.3 | 0.6 | 0.9 | 1.1 | 1.5 | 1.6 |

Table 2: Length of tomato cv. Red Robin roots over time.

| Tomato seed number | ------------------------------------Root length (mm) ------------------------------------ | | | | | | | | | |
| --- | --- | --- | --- | --- | --- | --- | --- | --- | --- | --- |
|  | Day 0 | Day 3 | Day 4 | Day 5 | Day 6 | Day 7 | Day 8 | Day 9 | Day 10 | Day 11 |
| 1 | 0 | 0 | 0 | 0.3 | 0.9 | 1.3 | 2 | 2.1 | 3 | 3.1 |
| 2 | 0 | 0 | 0 | 0 | 0 | 0 | 0 | 0.2 | 0.5 | 1.4 |
| 3 | 0 | 0 | 0 | 0 | 0 | 0.3 | 0.7 | 1.8 | 2.8 | 2.9 |
| 4 | 0 | 0 | 0 | 0 | 0 | 0 | 0 | 0 | 0.2 | 0.6 |
| 5 | 0 | 0 | 0 | 0 | 0.3 | 1.2 | 1.8 | 2.6 | 3.5 | 3.6 |
| 6 | 0 | 0 | 0 | 0 | 0.1 | 0.4 | 0.6 | 1.1 | 1.4 | 1.9 |
| 7 | 0 | 0 | 0 | 0.3 | 0.5 | 1 | 1.4 | 1.6 | 2 | 1.9 |
| 8 | 0 | 0 | 0 | 0.1 | 0.3 | 0.8 | 1.1 | 1.5 | 1.8 | 2 |
| 9 | 0 | 0 | 0 | 0 | 0 | 0 | 0 | 0 | 0.2 | 0.2 |
| 10 | 0 | 0 | 0 | 0 | 0.1 | 0.4 | 0.8 | 1.6 | 2.4 | 2.4 |
| 11 | 0 | 0 | 0 | 0 | 0.1 | 0.1 | 0 | 0.7 | 1.7 | 2 |
| 12 | 0 | 0 | 0 | 0.3 | 0.9 | 1.7 | 2.1 | 2.7 | 2.9 | 3 |
| 13 | 0 | 0 | 0 | 0 | 0 | 0 | 0 | 0.6 | 1.6 | 2.3 |
| Average | 0 | 0 | 0 | 0.1 | 0.2 | 0.6 | 0.8 | 1.3 | 1.8 | 2.1 |
| Standard deviation | 0 | 0 | 0 | 0.1 | 0.3 | 0.6 | 0.8 | 0.9 | 1.1 | 1.0 |
