# Supplementary material for: Germination and seedling establishment for hydroponics: The benefit of slant boards
Source: PLoS One. 2022 Oct 5;17(10):e0275710. doi: 10.1371/journal.pone.0275710 (PMC9534409; doi:10.1371/journal.pone.0275710)
Supplement: Attachment — Submitted filename: PONE-D-22-22878_reviewer.doc [file pone.0275710.s003.doc]

**PLOS ONE**

**Germination and seedling establishment for deep-flow hydroponics: The benefit of slant boards**

--Manuscript Draft--

| **Manuscript Number:** | PONE-D-22-22878 |
| --- | --- |
| **Article Type:** | Lab Protocol |
| **Full Title:** | Germination and seedling establishment for deep-flow hydroponics: The benefit of slant boards |
| **Short Title:** | Germination slant boards |
| **Corresponding Author:** | Noah James Langenfeld, B.S. Utah State University  Logan, Utah UNITED STATES |
| **Keywords:** | Seed germination; hydroponics; slant board; germination paper; deep-flow |
| **Abstract:** | Germination and seedling establishment for transplanting into hydroponics often uses porous substrates, but fine roots grow into these substrates, and they cannot be removed without damaging roots. Seedlings transplanted without removal of substrates can cause interactions with solution chemistry or addition of particulates to the nutrient solution. Germination of seeds on slant boards is clean, uniform, and reduces the time to transplanting. Slant boards facilitate long roots, which maximize exposure of the primary root to the nutrient solution after transplanting. The “boards” are made from thin acrylic or polycarbonate sheets with germination paper on top. Seeds are held in place by covering with thin paper before vertical placement of the boards in a container. Four to twelve days later, the seedlings with long roots can be removed from the paper without damage and transplanted into the hydroponic system. Here we describe slant board construction and procedures for rapid germination and transplanting. |
| **Order of Authors:** | Noah James Langenfeld, B.S. |
|  | Bruce Bugbee |
| **Additional Information:** |  |
| **Question** | **Response** |
| **Financial Disclosure**  Enter a financial disclosure statement that describes the sources of funding for the work included in this submission. Review the [submission guidelines](http://journals.plos.org/plosone/s/submission-guidelines#loc-financial-disclosure-statement) for detailed requirements. View published research articles from [*PLOS ONE*](http://journals.plos.org/plosone/) for specific examples.  This statement is required for submission and **will appear in the published article** if the submission is accepted. Please make sure it is accurate. | This research was supported by the Utah Agricultural Experiment Station, Utah State University, and approved as journal paper number 9600; NASA, Center for the Utilization of Biological Engineering in Space (grant number NNX17AJ31G). The funders had and will not have a role in study design, data collection and analysis, decision to publish, or preparation of the manuscript. |

*Powered by Editorial Manager® and ProduXion Manager® from Aries Systems Corporation*

| **Unfunded studies**  Enter: *The author(s) received no specific funding for this work.*  **Funded studies**  Enter a statement with the following details:   - Initials of the authors who received each award - Grant numbers awarded to each author - The full name of each funder - URL of each funder website - Have or will the sponsors or funders play any role in the study design, data collection (when applicable) and analysis, decision to publish, or preparation of the manuscript? - **NO** - Include this sentence at the end of your statement: *The funders had and will not have a role in study design, data collection and analysis, decision to publish, or preparation of the manuscript.* - **YES** - Specify the role(s) played. |  |
| --- | --- |
| * typeset |  |
| **Competing Interests**  Use the instructions below to enter a competing interest statement for this submission. On behalf of all authors, disclose any [competing interests](http://journals.plos.org/plosone/s/competing-interests) that could be perceived to bias this work—acknowledging all financial support and any other relevant financial or non- financial competing interests.  This statement is required for submission and **will appear in the published article** if the submission is accepted. Please make sure it is accurate and that any funding sources listed in your Funding Information later in the submission form are also declared in your Financial Disclosure statement.  View published research articles from  [*PLOS ONE*](http://journals.plos.org/plosone/) for specific examples. | The authors have declared that no competing interests exist. |

*Powered by Editorial Manager® and ProduXion Manager® from Aries Systems Corporation*

| **NO authors have competing interests**  Enter: *The authors have declared that no competing interests exist.*  **Authors with competing interests**  Enter competing interest details beginning with this statement:  *I have read the journal's policy and the authors of this manuscript have the following competing interests: [insert competing interests here]* |  |
| --- | --- |
| * typeset |  |
| **Ethics Statement**  Enter an ethics statement for this submission. This statement is required if the study involved:   - Human participants - Human specimens or tissue - Vertebrate animals or cephalopods - Vertebrate embryos or tissues - Field research   Write "N/A" if the submission does not require an ethics statement.  General guidance is provided below. Consult the [submission guidelines](http://journals.plos.org/plosone/s/submission-guidelines#loc-guidelines-for-specific-study-types) for detailed instructions. **Make sure that all information entered here is included in the Methods section of the manuscript.** | N/A |

*Powered by Editorial Manager® and ProduXion Manager® from Aries Systems Corporation*

| **Format for specific study types**  **Human Subject Research (involving human participants and/or tissue)**   - Give the name of the institutional review board or ethics committee that approved the study - Include the approval number and/or a statement indicating approval of this research - Indicate the form of consent obtained (written/oral) or the reason that consent was not obtained (e.g. the data were analyzed anonymously)   **Animal Research (involving vertebrate animals, embryos or tissues)**   - Provide the name of the Institutional Animal Care and Use Committee (IACUC) or other relevant ethics board that reviewed the study protocol, and indicate whether they approved this research or granted a formal waiver of ethical approval - Include an approval number if one was obtained - If the study involved *non-human primates*, add *additional details* about animal welfare and steps taken to ameliorate suffering - If anesthesia, euthanasia, or any kind of animal sacrifice is part of the study, include briefly which substances and/or methods were applied   **Field Research**  Include the following details if this study involves the collection of plant, animal, or other materials from a natural setting:   - Field permit number - Name of the institution or relevant body that granted permission |  |
| --- | --- |
|  |  |
| **Data Availability**  Authors are required to make the data underlying their research findings fully available, without restriction.  If the manuscript reports sample data generated using the protocol, the data underlying those results must be made available at the time of publication. A Data | Yes - sample data are fully available without restriction |

*Powered by Editorial Manager® and ProduXion Manager® from Aries Systems Corporation*

| Availability Statement describing where the data can be found is required at submission. When legal or ethical requirements prohibit public data sharing, authors must indicate how researchers may obtain access to the data. See the [PLOS Data Policy](https://journals.plos.org/plosone/s/data-availability) and [FAQ](https://journals.plos.org/plosone/s/data-availability#loc-faqs-for-data-policy) for detailed information.  A Data Availability Statement describing where the data can be found is required at submission. Your answers to this question constitute the Data Availability Statement and **will be published in the article**, if accepted. |  |
| --- | --- |
| **Important:** Stating ‘data available on request from the author’ is not sufficient. If your data are only available upon request, select ‘No’ for the first question and provide justification in the text box. |  |
| For protocols without sample data or results, you must state this in your Data Availability Statement.  Do the authors confirm that EITHER the protocol does not report results OR that sample data will be made available? |  |
| **Describe where the data may be found in full sentences. If you are copying our sample text, replace any instances of XXX with the appropriate details.**   - If the data are **held or will be held in a public repository**, include URLs, accession numbers or DOIs. If this information will only be available after acceptance, indicate this by ticking the box below. For example: *All XXX files are available from the XXX database (accession number(s) XXX, XXX.*). - If the data are all contained **within the manuscript and/or Supporting Information files**, enter the following: *All relevant data are within the* | All relevant data are within the manuscript and its Supporting Information files. |

*Powered by Editorial Manager® and ProduXion Manager® from Aries Systems Corporation*

| *manuscript and its Supporting Information files.*   - If neither of these applies but you are able to provide **details of access elsewhere**, with or without limitations, please do so. For example:   *Data cannot be shared publicly because of [XXX]. Data are available from the XXX Institutional Data Access / Ethics Committee (contact via XXX) for researchers who meet the criteria for access to confidential data.*  *The data underlying the results presented in the study are available from (include the name of the third party and contact information or URL).*   - This text is appropriate if the data are owned by a third party and authors do not have permission to share the data.   * typeset |  |
| --- | --- |
| Additional data availability information: |  |

*Powered by Editorial Manager® and ProduXion Manager® from Aries Systems Corporation*

[Click here to access/download;Manuscript;Slant board 12 August 2022.docx](https://www.editorialmanager.com/pone/download.aspx?id=31664287&guid=40b39eda-f308-4415-99d8-32ab269113c2&scheme=1)

1 Germination and seedling establishment for deep-flow hydroponics: The benefit of slant boards
2 Germination slant boards
3 Noah James Langenfeld^1^, Bruce Bugbee^1^ 4
5 ^1^Crop Physiology Laboratory, Department of Plants, Soils, and Climate, Utah State University,
6 Logan, Utah, United States of America 7
8 * Corresponding author
9 E-mail: [noah.langenfeld@usu.edu](mailto:noah.langenfeld@usu.edu) (NJL) 10

11

12

# 13 Associated content

14 <http://dx.doi.org/10.17504/protocols.io.5jyl89b86v2w/v2> 15

16

17

18

# 19 Abstract

20 Germination and seedling establishment for transplanting into hydroponics often uses
21 porous substrates however, the fine roots of seedlings grow into these substrates, and they cannot be removed without
22 damaging these roots. Also, the seedlings transplanted without removal of substrates can cause alterations in
23 solution chemistry or addition of particulates to the nutrient solution. Germination of seeds on
24 slant boards is clean, uniform, and reduces the time to transplanting. Slant boards facilitate development of long
25 roots, which maximizes the exposure of the primary root to the nutrient solution after transplanting.
26 The “boards” are made from thin acrylic or polycarbonate sheets with germination paper on top.
27 Seeds are held in place by covering with thin paper before vertical placement of the boards in the
28 container. Four to twelve days later, the seedlings with long roots can be removed from the paper
29 without damage and transplanted into the hydroponic system. Here we describe the slant board
30 construction and procedures for rapid germination and transplanting for deep-flow hydroponics.

# 31 Introduction

32 Hydroponics is often used as a research tool in plant nutrition studies because of the
33 precise control of root-zone conditions and detailed monitoring of root health. Liquid culture
34 hydroponics does not use a solid substrate and therefore has no adsorption and desorption of
35 nutrients, which can complicate nutritional studies. These systems work well with the mass-
36 balance approach as nutrients are either in the solution or in the plant [1].
37 Rapid, uniform germination is essential for research in hydroponic systems. Few seeds
38 can germinate submerged in water [2] and thus cannot be planted directly into the nutrient
39 solution in liquid hydroponics. Submersion in water inhibits oxygen diffusion across seed
40 membranes and prevents germination [3].
41 For maximum uniformity, seeds should be germinated separately and transplanted into
42 the main system when roots are long enough to be in contact with the nutrient solution.
43 (Fig 1). Reduced time for germination is beneficial so plants can
44 be subjected to treatments in hydroponics systems for a larger percentage of their lifecycle.
45 **Fig 1.** Side-view diagram of seedling transplanted into a hydroponic tank. The root must be long
46 enough to span the 1 cm air gap and have ample access to the solution immediately after
47 transplanting.
48 Germination methods utilizing a solid substrate such as peat moss, coco coir, perlite, or
49 vermiculite [4] are often used, but transplanting requires removing all the substrate from the
50 roots to avoid its introduction into the hydroponic system. This process damages fine root hairs
51 and stunts seedling growth following transplanting.
52 Seeds germinated in inert mineral wool or floral foam can be transplanted directly into
53 hydroponic systems as plugs, but they can easily be flooded if the media contacts the solution
54 after transplanting. Germinating on a rolled paper towel is common, but germination is not as
55 uniform, and the density of the seeds is often excessive.
56 Transparent boxes with germination paper on the bottom are widely used for germination
57 testing, but root and seedling growth are not straight, which makes transplanting difficult.
58 Germination in porous ceramic media (calcined clay) facilitates removal of roots from the
59 substrate, but this media does not allow selection of plants with long roots, and it is time
60 consuming to rinse the particles from the root surfaces without damaging the roots. This product
61 was historically marketed under the trade name ‘Arcillite®’, but it is now sold under the trade
62 name ‘Profile® Greens Grade™’ ([https://www.profileevs.com/products/soil-](https://www.profileevs.com/products/soil-amendments/profile-porous-ceramic-ppc)
63 [amendments/profile-porous-ceramic-ppc](https://www.profileevs.com/products/soil-amendments/profile-porous-ceramic-ppc)). For many years it was used for plant growth studies
64 on the International Space Station because the roots can be removed from the media for analysis

65	[5].

66 Jones and Cobb (1963) appear to be the first to use the slant board method to rapidly
67 germinate seeds with long and straight roots. Their tests showed high germination rates for seeds
68 on germination paper placed on an acrylic board at a 67° angle and covered with a single layer of
69 cellulose tissue. We have adopted this method for the rapid germination and selection of the
70 best seedlings for transplanting to hydroponic culture.
71 *Slant board system*
72 A slant board system has five component parts: the board, germination paper, thin
73 absorbent paper, an exterior container, and a positioning device.
74 The **slant board** is a thin (2 mm) piece of acrylic or polycarbonate cut to about 15 cm tall
75 and 30 cm wide. Glass should be avoided due to the safety hazard from potential breakage and
76 wood is not inert.
77 **Germination paper** is dense enough to prevent root penetration, has excellent hydraulic
78 conductivity, is pH adjusted to a biological range (6-7), and is free from dyes that can influence
79 root growth. It is sold by many suppliers (e.g., Seedburo, Anchor Paper, Nasco) and costs about
80 1 USD (2022) per 10 by 14-inch sheet. Paper color does not make a difference in germination
81 quality, but fine root hairs are easier to monitor on blue colored germination paper [7] (Fig 2).
82 **Fig 2.** Planting of lettuce (*Latuca sativa*) seeds on blue germination blotter paper.
83 The **thin absorbent paper** is used to cover seeds. We have found single-ply ‘Kimwipes’
84 to work well because the roots are visible through the paper. Consumer-grade paper towels also
85 work well and are widely available. Most roots do not penetrate the paper, but we have seen
86 wheat roots grow through and become difficult to remove (Fig 3). A second piece of germination
87 paper may be used in place of the thin paper in these situations. Tissue paper (e.g. Kleenex®)
88 should not be used because it breaks when water is added to the system.
89 **Fig 3.** Example of the growth of wheat roots through the thin absorbent paper. It is difficult to
90 separate the roots from the paper when this occurs. In this case, an additional piece of
91 germination paper is recommended in place of the thin absorbent paper.
92 The **exterior container** can be any type of inert box that holds 2 to 3 cm of water and is
93 large enough to hold all slant boards. Plastic boxes are lightweight, inert, and available in a
94 variety of shapes and sizes. The container may be clear or opaque, but opaque containers may
95 help limit direct light exposure to fragile roots.
96 The **positioning device** is used to hold the slant boards at angles of 70° – 80° in the
97 exterior container and should be plastic to avoid corrosion. This can be milled to size with a table
98 saw from a thick piece of acrylic (Fig 4) or polycarbonate. Aluminum (Al) should be avoided as
99 it can release Al into the solution.
100 **Fig 4.** Acrylic holder for slant boards.
101 *Seedling variability and environmental conditions*
102 More seeds should be planted than needed to ensure selection for uniform roots. We
103 recommend overseeding by 100%. The cost of overseeding is outweighed by the ability to select
104 for uniformity.
105 A complete nutrient solution is not necessary as seeds have enough nutrients for the first
106 few days of growth and it quickly leads to undesirable algal growth on the surfaces exposed to
107 light. However, water with about 40 ppm (1 mM) calcium (Ca) should be used for germination
108 because Ca promotes healthy growth of seedling roots. Calcium nitrate can be added to purified
109 water to achieve this Ca concentration. The water level in the plastic container should be
110 maintained so that the level is about 2 cm above the bottom of the germination paper. Levels
111 lower than 2 cm may lead to desiccation and levels higher than 2 cm result in excessive water at
112 the seed level and reduced oxygen transfer to the seeds.
113 Light is required for germination of some species, and it keeps hypocotyls short after the
114 cotyledons emerge. Exposure to direct sunlight should be avoided, but indirect sunlight and
115 supplemental lighting help with early photosynthesis of young seedlings. Light levels in
116 laboratories are usually less than 20 µmol m^-2^ s^-1^, which is inadequate to keep the seedling short
117 and can lead to hypocotyl elongation [8]. We have used small, low-cost LED fixtures to provide
118 100 to 200 µmol m^-2^ s^-1^ over slant boards on a laboratory bench (Fig 5). These are available from
119 multiple sources (See examples in this home horticulture video:
120 <https://www.youtube.com/watch?v=m3rBpc7ue74>).
121 **Fig 5.** Germination slant boards under LED lights. The photosynthetic photon flux density
122 should be about 100 to 200 µmol m^-2^ s^-1^ to provide adequate light to reduce excessive hypocotyl
123 extension.
124 The temperature should be 20 to 25 °C. Higher temperatures can increase germination
125 rates but decrease the solubility of oxygen in water and can decrease root elongation uniformity;
126 lower temperatures often increase time to germination.
127 Not all seeds germinate and grow at the same rate. We have found that lettuce (*Latuca*
128 *sativa*), wheat (*Triticum aestivum*) and soybean (*Glycine max*) germinate quickly and are ready
129 for transplanting in less than seven days. Tomato (*Solanum lycopersicum*) and pepper (*Capsicum*
130 *annuum*) require more than seven days. Some species with large seeds, such as corn (*Zea mays*)
131 and cucumber (*Cucumis sativus*), require additional moisture for germination. Placing a second
132 piece of germination paper on top of the seeds can help to maintain moisture for these species
133 (Fig 6 and 7).
134 **Fig 6.** Side view of cucumbers on slant board with an additional piece of germination paper on
135 top of the thin absorbent paper.
136 **Fig 7.** Cucumber seedlings on a slant board with two layers of germination paper. The roots of
137 cucumber seedlings branch more than other species such as lettuce and tomato.
138 Spinach (*Spinacia oleracea*) is difficult to germinate because of a hard seed coat and
139 germination inhibitors [9]. Following the method of Katzman et al. (2001), we significantly
140 improved germination with a 4-hr pretreatment in a continuously-stirred solution of 0.5% sodium
141 hypochlorite (a 1:10 dilution of standard commercial bleach), followed by a 15-hr rinse in
142 continuously aerated distilled water to remove bleach residue. Seeds were then placed in
143 germination boxes with germination paper soaked with 0.3% hydrogen peroxide (a 1:10 dilution
144 of standard commercial hydrogen peroxide) to signal germination. It was also reported that lowering
145 temperature from 30 to 18 °C increased germination. This method was used to increase
146 germination of ‘Bloomsdale’ and ‘Melody’ spinach cultivars from 20 to over 90% compared to
147 non-treated controls. Both bleach and hydrogen peroxide are beneficial for maximum
148 germination, however, our protocol recordedthat hydrogen peroxide alone improves germination more than
149 bleach. Wojtyla et al. (year reference missing?) reviewed the value of hydrogen peroxide as a signaling molecule in
150 promoting germination. As soon as the radicle emerges, the seeds should be transferred to slant
151 boards for root elongation.
152 *Transplanting into hydroponic systems*
153 Seeds should be transplanted as soon as the roots are adequately long. Growth rate is
154 diminished if seedlings are left on the slant board because they do not have access to nutrients.
155 In this study it was observed that root elongation starts to decrease after about seven days for lettuce and 12
156 days for tomato.
157 The seedlings were transplanted into neoprene cloning collars, which are widely sold online
158 (Amazon.com, Walmart.com). These collars come with a radial slit in one side, gently hold
159 young seedlings above the nutrient solution, and minimize root growth into the material (Fig 8).
160 These collars can be directly placed in a rigid cover (Fig 9) or can be floated on the nutrient
161 solution surface.
162 **Fig 8.** Neoprene cloning collar with lettuce seedling. The top of the collar has been painted white
163 to reduce light penetration into the nutrient solution, reduce temperature, and to reflect light back
164 to leaves for maximum growth. A radial slit in the neoprene collar runs between the thumb and
165 the plant.
166 **Fig 9.** Lettuce seedling in neoprene collar placed in rigid cover above nutrient solution surface
167 about 7 days after transplanting.
168 Germinating seeds on a slant board facilitates rapid growth of long, straight roots.
169 Uniform seedlings can be selected and easily removed for transplanting without damaging root
170 hairs. All components except the papers are reusable and the system is easily scalable for
171 multiple seed sizes and species.

# 172 Materials and methods

173 The protocol described in this peer-reviewed article is published on protocols.io,
174 <http://dx.doi.org/10.17504/protocols.io.5jyl89b86v2w/v2> and is included for printing as S1 File
175 with this article.

# 176 Expected Results

177 Use of a slant board results in rapid root elongation and easy transplanting four to seven
178 days following germination for lettuce (Fig 10) and nine to eleven days after germination for tomato.
179 Seedling vigor is reduced after this time as nutrients stored in plant seeds gets depleted.
180 Transplanting into nutrient solution should occur around day five for lettuce and day 10 for
181 tomato to maintain exponential crop growth (Fig 11). Lettuce that is transplanted into treatments
182 seven days after seeding and is harvested 35 days after seeding means 80% of the lifecycle
183 occurs when exposed to treatment conditions.
184 **Fig 10.** Slant boards with germination blotter paper, lettuce seedlings, and thin paper over the
185 seeds from day zero to day seven post seeding. The thin paper can be removed to expose roots
186 for inspection. Over-seeding helps for selection of uniform roots.
187 **Fig 11.** Example of rapid and excessive growth of lettuce (cv. Grand Rapids) and tomato (cv.
188 Red Robin) roots over time. Root elongation rate starts to decrease after roots are 20 to 30 mm
189 long. Error bars represent standard deviation, n=13. There is a large variability among seedlings.
190 Overplanting by a factor of at least two allows for selection of the most vigorous seedlings.

# 191 References

192 1.	Bugbee B. Nutrient management in recirculating hydroponic culture. Acta Hortic. 2004; 193	99–112. doi:10.17660/ActaHortic.2004.648.12

194	2.	Morinaga T. Germination of seeds under water. American Journal of Botany. 1926;13: 195	126–140. doi:10.2307/2435353

196	3.	Dasberg S, Mendel K. The Effect of Soil Water and Aeration on Seed Germination. J Exp 197	Bot. 1971;22: 992–998. doi:10.1093/jxb/22.4.992

198	4.	Kim H-J, Yang T, Lin M-Y, Langenhoven P. Plant propagation for successful hydroponic 199	production. Acta Hortic. 2018; 109–116. doi:10.17660/ActaHortic.2018.1212.22

200 5.	Flemming C, Seck S, Massa G, Hummerick M, Wheeler R. From Kennedy, to beyond:
201 Growing plants in Space. John F. Kennedy Space Center: NASA; 2012. Available:
202 https://ntrs.nasa.gov/api/citations/20120015738/downloads/20120015738.pdf
203 6.	Jones L, Cobb R. A technique for increasing the speed of laboratory germination.
204 Proceedings of the Association of Official Seed Analysts. 1963;53: 144–160.
205 7.	Booth DT, Griffith LW. Measuring Post-Germination Growth. Journal of Range 206	Management. 1994;47: 503. doi:10.2307/4003006

207	8.	van Gelderen K, Kang C, Pierik R. Light Signaling, Root Development, and Plasticity. 208	Plant Physiol. 2018;176: 1049–1060. doi:10.1104/pp.17.01079

209 9.	Hartmann HT, Davies FT, Geneve RL, Wilson SB. Hartmann & Kester’s plant propagation:
210 principles and practices. Ninth edition. NY, NY: Pearson; 2018.
211 10.	Katzman LS, Taylor AG, Langhans RW. Seed Enhancements to Improve Spinach 212	Germination. HortSci. 2001;36: 979–981. doi:10.21273/HORTSCI.36.5.979

213

# 214 Supporting information

215 **S1 File. Protocol for slant board method from protocols.io.**
216 **S2 File. Root length measurements from Figure 11 in main text.**

Figure 1	[Click here to access/download;Figure;Figure 1.tif](https://www.editorialmanager.com/pone/download.aspx?id=31664449&guid=9688af61-cd17-4f7f-88ca-a8020fdf22c7&scheme=1)

Figure 2	[Click here to access/download;Figure;Figure 2.tif](https://www.editorialmanager.com/pone/download.aspx?id=31664451&guid=ad215cfb-07ed-4de4-b1db-6ee70d0d59fa&scheme=1)

Figure 3	[Click here to access/download;Figure;Figure 3.tif](https://www.editorialmanager.com/pone/download.aspx?id=31664455&guid=0d3d15c9-d218-4478-a377-c435f9234d48&scheme=1)

Figure 4	[Click here to access/download;Figure;Figure 4.tif](https://www.editorialmanager.com/pone/download.aspx?id=31664457&guid=d60e2ef2-19d4-4415-b883-34e440716b45&scheme=1)

Figure 5	[Click here to access/download;Figure;Figure 5.tif](https://www.editorialmanager.com/pone/download.aspx?id=31664458&guid=8791f136-394c-416f-9094-0084d8cdb145&scheme=1)

Figure 6	[Click here to access/download;Figure;Figure 6.tif](https://www.editorialmanager.com/pone/download.aspx?id=31664459&guid=a83ba2d2-4280-4ba4-8a23-823d393de13c&scheme=1)

Figure 7	[Click here to access/download;Figure;Figure 7.tif](https://www.editorialmanager.com/pone/download.aspx?id=31664460&guid=b3845be5-d0d3-46c5-a2e6-ba0f3cef8a63&scheme=1)

Figure 8	[Click here to access/download;Figure;Figure 8.tif](https://www.editorialmanager.com/pone/download.aspx?id=31664461&guid=77a58c07-f338-45c1-8595-dc6b4a8e2343&scheme=1)

Figure 9	[Click here to access/download;Figure;Figure 9.tif](https://www.editorialmanager.com/pone/download.aspx?id=31664462&guid=c29c0b48-dbdc-4bec-a3e8-b7cbbb4065d7&scheme=1)

Figure 10	[Click here to access/download;Figure;Figure 10.tif](https://www.editorialmanager.com/pone/download.aspx?id=31664463&guid=baaa4f7e-a676-4d86-8cac-d91196c309b2&scheme=1)

Figure 11	[Click here to access/download;Figure;Figure 11.tif](https://www.editorialmanager.com/pone/download.aspx?id=31664464&guid=5ee672f9-b7fe-468a-927a-ec0294d74659&scheme=1)

Supporting Information 1

[Click here to access/download](https://www.editorialmanager.com/pone/download.aspx?id=31664466&guid=a188a8c5-1383-43c5-bc3e-247474da7db3&scheme=1)

[**Supporting Information** S1 Protocol.pdf](https://www.editorialmanager.com/pone/download.aspx?id=31664466&guid=a188a8c5-1383-43c5-bc3e-247474da7db3&scheme=1)

Supporting Information 2

## [Click here to access/download](https://www.editorialmanager.com/pone/download.aspx?id=31664468&guid=d3d0c1ac-e967-44a2-ad27-fd92f9cb7044&scheme=1)

[**Supporting Information** S2 File.docx](https://www.editorialmanager.com/pone/download.aspx?id=31664468&guid=d3d0c1ac-e967-44a2-ad27-fd92f9cb7044&scheme=1)
