# Supplementary material for: Germination and seedling establishment for hydroponics: The benefit of slant boards
Source: PLoS One. 2022 Oct 5;17(10):e0275710. doi: 10.1371/journal.pone.0275710 (PMC9534409; doi:10.1371/journal.pone.0275710)
Supplement: Attachment — Submitted filename: Rebuttal 16 September 2022.docx [file pone.0275710.s004.docx]

Dear editors and reviewers,

Thank you for your thoughtful comments and feedback on our seed germination manuscript. We have included a point-by-point discussion detailing our changes per your suggestions below:

**Academic editor:**

We added two sentences (line 103) describing how root entanglement was not observed regardless of the high planting density. This is an important consideration; thank you for your comment.

We added two sentences (line 111) discussing how frequently water must be added to maintain the 2 cm depth. Thank you for this comment.

References have been checked to ensure uniformity.

**Reviewer 1:**

The slant board is simply larger than the blue colored paper to leave room for labeling. The positioning of the seeds is described in step 4 of the protocol on protocols.io.

Two sentences have been added discussing seeding density and root entanglement. See line 111.

We believe the protocol is described in sufficient detail on protocols.io. Please see this for the detailed protocol.

**Reviewer 2:**

Thank you for your edits in the abstract. We have included most of these to make the abstract stronger. We also broaden the title and last sentence of the abstract to focus on hydroponics in general as opposed to specifically deep-flow.

Line 69 changed from plural to singular method.

We added two fundamental references describing the effects of temperature on seed germination and dissolved oxygen. Thank you for this comment.

We simplified the sentence about hydrogen peroxide alone being better than bleach alone.

We have fixed the missing Wojtyla reference.

We have changed our “We have…” statements to be more objective per your recommendation.

One line 183, numbers above 10 should be numbers and numbers below 10 should be spelled out. We edited the following sentence per your recommendation.

Sincerely,

Noah J. Langenfeld and Bruce Bugbee
